# Two Shorter Variants of the Proline-Rich Antimicrobial Peptide B7-005 Scaffold Active Against Clinical Isolates of *Pseudomonas aeruginosa* and *Staphylococcus aureus*

**DOI:** 10.3390/antibiotics15040412

**Published:** 2026-04-18

**Authors:** Giacomo Cappella, Adriana Di Stasi, Clelia Cortese, Luisa Torrini, Agnese D’Amore, Virginia Niccolini, Luigi de Pascale, Bruno Casciaro, Mario Mardirossian, Alessandro Pini, Maria Luisa Mangoni, Marco Scocchi

**Affiliations:** 1 Department of Biochemical Sciences “A. Rossi Fanelli”, Laboratory Affiliated to Istituto Pasteur Italia-Fondazione Cenci Bolognetti, Sapienza University of Rome, 00185 Rome, Italy; giacomo.cappella@uniroma1.it (G.C.); bruno.casciaro@uniroma1.it (B.C.); 2 Department of Life Sciences, University of Trieste, 34127 Trieste, Italy; adriana.distasi@units.it (A.D.S.); luigi.depascale@phd.units.it (L.d.P.); mmardirossian@units.it (M.M.); 3 Department of Medical Biotechnology, University of Siena, 53100 Siena, Italy; clelia.cortese@student.unisi.it (C.C.); virginia.niccolini@unisi.it (V.N.); alessandro.pini@unisi.it (A.P.); 4 Department of Molecular Medicine, Sapienza University of Rome, 00185 Rome, Italy; luisa.torrini@uniroma1.it; 5 Clinical Pathology Unit, Azienda Ospedaliera Universitaria Senese, via Mario Bracci, 53100 Siena, Italy; 6 SetLance Srl, via Fiorentina 1, 53100 Siena, Italy

**Keywords:** antimicrobial peptides, antibiotic resistance, drug discovery, ESKAPE pathogens, biocompatibility, biostability

## Abstract

**Background/Objectives:** Developing novel strategies to combat respiratory infections caused by multidrug-resistant “priority pathogens” like the ESKAPEE *Pseudomonas aeruginosa* and *Staphylococcus aureus* is an urgent priority. **Methods:** We investigated two shortened variants of the proline-rich antimicrobial peptide (PrAMP) B7-005, B7-006 (15-mer) and B7-007 (13-mer). Evaluation included MIC assays against laboratory and clinical multidrug-resistant isolates, mechanistic studies of membrane permeabilization, cytotoxicity testing on BEAS-2B bronchial epithelial cells, and proteolytic stability assays in human elastase and sputum. **Results:** Despite their reduced size, lower positive charge, and decreased proline content, both variants retained full antimicrobial activity against clinical pathogens with consistent MIC values ≤ 25 µM. These variants exhibit membrane permeabilization in *P. aeruginosa* but may also relay on a hybrid mode of action involving also intracellular targets. Notably, B7-006 and B7-007 displayed low cytotoxicity compared to the lytic peptide BMAP-18. While B7-007 showed greater susceptibility to proteolytic degradation than its parent B7-005, it preserved partial integrity during the initial hours of exposure. **Conclusions:** Overall, these findings demonstrate that the B7 scaffold tolerates substantial truncation while preserving potency and selectivity, identifying a minimal 13-amino-acid active core. This work provides critical insights into structure–activity relationships and supports the development of compact, mechanistically versatile antimicrobial peptides to address the growing threat of multidrug-resistant respiratory pathogens.

## 1. Introduction

The growing need for alternatives to traditional antibiotics has driven increasing interest in antimicrobial peptides (AMPs) as promising therapeutic candidates, given their potential to improve public health outcomes [1,2,3]. This need is particularly evident in the field of respiratory infections, which remain a major cause of morbidity and mortality worldwide and are progressively getting complicated by the spread of multidrug-resistant (MDR) pathogens [4]. Among the most prevalent and clinically challenging bacteria responsible for pulmonary infections are *Pseudomonas aeruginosa* and *Staphylococcus aureus*, frequently associated with hospital-acquired pneumonia and chronic lung diseases such as cystic fibrosis [5,6]. The emergence of antibiotic-resistant strains of these species has further narrowed the already limited panel of therapeutic options, reinforcing the urgency of developing novel antimicrobial strategies [7].

AMPs are short, cationic peptides typically composed of 10–50 amino acids, approximately half of which are hydrophobic [1,8]. While most AMPs exert their bactericidal activity through disruption and permeabilization of the microbial membrane, a subset operates via intracellular mechanisms, targeting vital cellular functions without significantly compromising membrane integrity at microbicidal concentrations [9,10]. Among these, proline-rich antimicrobial peptides (PrAMPs) represent the most extensively characterized class [11,12]. PrAMPs, originating from largely different animals [9,13], share a conserved mode of action. They enter the bacterial cells by exploiting membrane bacterial transporters, such as SbmA, and bind to the exit tunnel of ribosomes, thus inhibiting protein synthesis [14,15]. This uptake-dependent mechanism confers selective activity by reducing toxicity on eukaryotic cells. However, it also limits their spectrum of activity to species expressing the necessary transport systems or otherwise able to internalize the peptides.

Numerous chemical modifications have been explored to optimize PrAMPs to broaden their spectrum of activity while preserving their specific mode of action on ribosomes, including lipid conjugation [16,17], sequence truncation [11,18], the incorporation of heterologous peptide segments or point mutations [19,20,21] and sequence modification [22,23,24]. B7-005 has emerged as a promising synthetic derivative of PrAMPs, displaying broad-spectrum activity that includes Gram-positive bacteria and ESKAPEE pathogens, even in the absence of SbmA-mediated uptake, likely due to its increased hydrophobicity [25,26]. The potential of B7-005 to eradicate these pathogens was evaluated in both planktonic and biofilm forms, revealing species-dependent bactericidal and anti-biofilm effects across all ESKAPEE pathogens [27]. B7-005’s mechanism of action also varied depending on the target microorganism, ranging from intracellular inhibition of protein synthesis without detectable membrane damage to different levels of membrane permeabilization [27].

During the synthesis and purification of B7-005, we observed the co-elution of a highly active species. Subsequent elucidation by mass spectrometry identified two shorter variants sharing the B7-005 scaffold, which we named B7-006 and B7-007.

Given the relevance of *P. aeruginosa* and *S. aureus* in respiratory infections, both of which were previously determined to be highly sensitive to the parental B7-005 [27], and the protease-rich environment of the inflamed lung, the development of peptide-based therapeutics requires not only antimicrobial activity against clinical and MDR isolates, but also resistance to lung-associated proteases and low cytotoxicity toward bronchial epithelial cells.

In this study, we aimed to further characterize the properties of B7-005 and evaluate whether its shorter variants could be equally effective. We assessed the antimicrobial effectiveness of B7-005, B7-006, and B7-007 against a panel of clinical *P. aeruginosa* and *S. aureus* isolates, including antibiotic-resistant strains, and their mode of action in comparison with the membranolytic α-helical peptide BMAP-18. Furthermore, we assessed their stability against proteolytic degradation (in human elastase and sputum) and their safety toward human bronchial epithelial cells. Our results highlight that the size of active PrAMP-derived peptides can be reduced to 13 amino acids, representing one of the shortest proline- and arginine-containing peptides known to date, while maintaining antimicrobial potency and protease stability without increasing cytotoxicity.

## 2. Results

### 2.1. Properties of B7-005 Variants

B7-006 and B7-007, identified as by-products during the synthesis and purification of B7-005, were characterized by mass spectrometry (Table 1).

B7-006 is a 15-mer peptide that lacks arginine at position 6 (Arg6), resulting in the loss of a positive charge. This modification could alter the peptide’s interactions with the bacterial membrane, potentially affecting its bactericidal mechanism.

Furthermore, B7-007 lacks proline at position 13 (Pro13) and arginine at position 14 (Arg14). These deletions not only decrease the peptide’s overall charge but may also significantly affect its natural properties. In particular, the removal of proline (an amino acid known to influence peptide conformation) could alter the three-dimensional arrangement of the molecule. With only two proline residues remaining, B7-007 no longer fulfils the current definition of a Proline-rich Antimicrobial Peptide (PrAMP) according to Welsh et al. [5,6] (cationic peptides with proline ≥25%). We thought these sequence modifications may provide valuable insight for structure-activity relationship studies, helping to elucidate how specific variations in the amino acid sequence influence antimicrobial activity, stability, and toxicity. Such analyses facilitate the identification of key molecular “motifs” that are essential for the peptide’s function.

### 2.2. Antimicrobial Activity and Bacterial Membrane Perturbation

Initially, we verified the antimicrobial activity of B7-006 and B7-007 on *Escherichia coli* ATCC 25922. The Minimum Inhibitory Concentrations (MIC) for both variants were found to be 1.56–3.12 µM, which were identical to or two-fold higher than those observed for B7-005. We then tested the peptides on two different strains of *P. aeruginosa*, confirming that both variants fully retained antimicrobial activity with negligible MIC variations (Table 2).

Afterwards, we investigated whether the two shortened variants, with their reduced charge and proline content, maintained the same mode of action of B7-005. Based on the MIC results, we assessed the peptides’ ability to permeabilize the cytoplasmic membrane of *E. coli* and *P. aeruginosa*.

Membrane integrity of bacteria exposed to B7-006 and B7-007 at their MICs was evaluated by measuring the uptake of propidium iodide (PI), a fluorescent marker of compromised cell membranes (Supplementary Figure S1).

**Figure 1:**
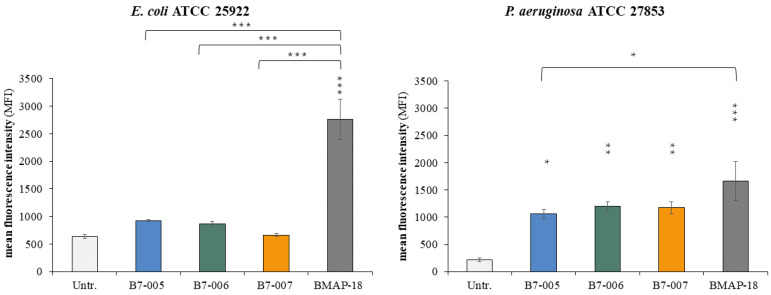
Membrane permeabilization assay on *E. coli* ATCC 25922 and *P. aeruginosa* ATCC 27853. Membrane-damaging activity was evaluated by flow cytometry. Permeabilization of the bacterial membrane was assessed by the uptake of propidium iodide (PI), a fluorescent marker for compromised membranes, after 1 h of exposure to the peptides. Peptides were used at their respective MICs against each bacterial species. The mean fluorescence intensity (MFI) of the population was measured. Error bars represent the standard deviations calculated from the average of at least three independent experiments (n ≥ 3). * *p* < 0.05, ** *p* < 0.01, *** *p* < 0.001 compared with the untreated sample or BMAP-18 (one-way ANOVA test followed by Tukey’s multiple comparisons test).

Consistent with the behaviour of the parental B7-005, neither B7-006 nor B7-007 significantly increased PI fluorescence in *E. coli*, indicating that the bacterial membranes remained largely intact (Figure 1). In contrast, the membranolytic peptide BMAP-18, used as a positive control [28], induced high levels of permeabilization under the same conditions. However, a distinct profile was observed in *P. aeruginosa*. While PrAMPs are traditionally considered non-lytic, previous studies demonstrated that B7-005 exhibits a significant ability to perturb the *P. aeruginosa* membrane, as well as that of *S. aureus*, while its effect on the protein synthesis of these pathogens is reduced compared to its action in *E. coli* [27].

We therefore analyzed whether the B7-006 and B7-007 variants exhibited a similar mechanism of action to B7-005. Results indicate that both B7-006 and B7-007, as well as B7-005, did not affect significantly the integrity of the *E. coli* membrane within a 1-h incubation (Figure 1). Our results confirm that B7-006 and B7-007 maintain this species-specific mechanism; both variants permeabilized *P. aeruginosa* membranes within 1 h to an extent comparable to B7-005. Although these permeabilization levels were lower than those of the highly lytic BMAP-18, they remain biologically significant (Figure 1).

Collectively, these data suggest that the B7 variants share the dual mode of action of B7-005. In *E. coli*, the activity appears primarily driven by a non-lytic mechanism of action likely relying on the inhibition of protein synthesis without membrane damage, whereas in *P. aeruginosa*, a hybrid mechanism may involve significant membrane destabilization alongside potential intracellular targeting.

Considering the similarity in this mode of action and the broad-spectrum of activity of B7-005 against *P. aeruginosa* and *S. aureus* [26], the three peptides were then evaluated against a panel of clinical isolates of these two species since they are the most representative pathogens involved in pulmonary infections: *P. aeruginosa* and *S. aureus.* The activity was compared to that of the peptide BMAP-18, which was previously reported to exhibit high activity against these species [28].

**Figure 2:**
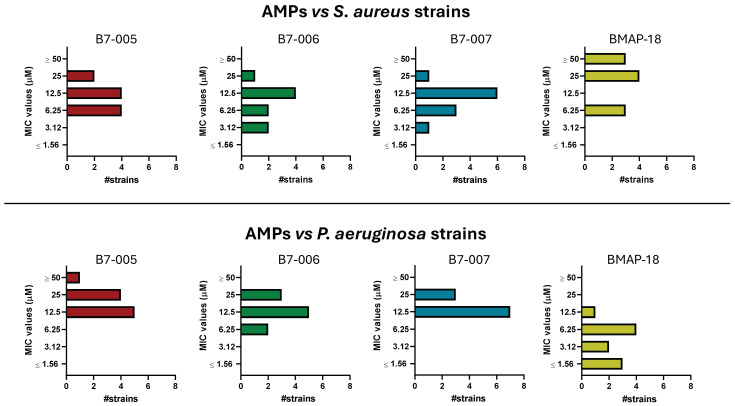
Distribution of MIC values for each peptide against different strains of *S. aureus* (**top panel**) and *P. aeruginosa* (**bottom panel**). The data are expressed as the number (indicated by #) of strains associated with each MIC value. Each graph represents a single peptide. The MIC values, expressed in µM, are the mode of at least three biological replicates (n ≥ 3). MIC was recorded after 18 h of incubation at 37 °C.

All three B7-derived AMPs demonstrated potent activity across all tested clinical isolates, with MIC values ranging from 3.12 to 25 µM for *S. aureus* and 6.25 to 25 µM for *P. aeruginosa* (Figure 2 and Supplementary Table S1). All variants exhibited consistent efficacy across diverse isolates. As shown in Figure 2, the MIC values followed a narrow distribution for each peptide, with most of the strains clustering within two or three adjacent concentration ranges. This highlights the high degree of consistency in the peptides’ antimicrobial performance (Figure 2). Specifically, the calculated MIC_90_ (the concentration required to completely inhibit the growth in 90% of isolates listed in Supplementary Table S1, i.e., 9 out of 10) was 25 µM for B7-005 and B7-006 against *S. aureus* isolates, while B7-007 showed slightly higher potency with an MIC_90_ 12.5 µM. Against *P. aeruginosa*, all three peptides maintained a consistent MIC_90_ 25 µM. Although the last five isolates of both species exhibited documented MDR phenotypes (Supplementary Tables S2 and S3), their MIC values remained within the same overall range as those of sensitive strains. While MICs for MDR *S. aureus* were occasionally up to two-fold higher than those observed for non-resistant strains, they did not deviate from the established potency range. Furthermore, no consistent increase in MIC values was observed among MDR *P. aeruginosa* strains. Overall, these data collectively demonstrate that the mechanism of action for B7 variants is not compromised by pre-existing resistance mechanisms, supporting their broad-spectrum utility against challenging clinical isolates.

The activity of ll three peptides were more consistent than BMAP-18, which was scarcely active against *S. aureus* (MIC_90_ = 50 µM), more active against *P. aeruginosa* strains (MIC_90_ = 6.25 µM), but exhibited high variability in sensitivity among strains (from 0.78 µM to more than 100 µM).

### 2.3. Evaluation of In Vitro Safety

Since the selected peptide variants displayed strong activity against clinically relevant pulmonary pathogens, we evaluated their potential cytotoxic effects on BEAS-2B human bronchial epithelial cells to assess their safety profile.

**Figure 3:**
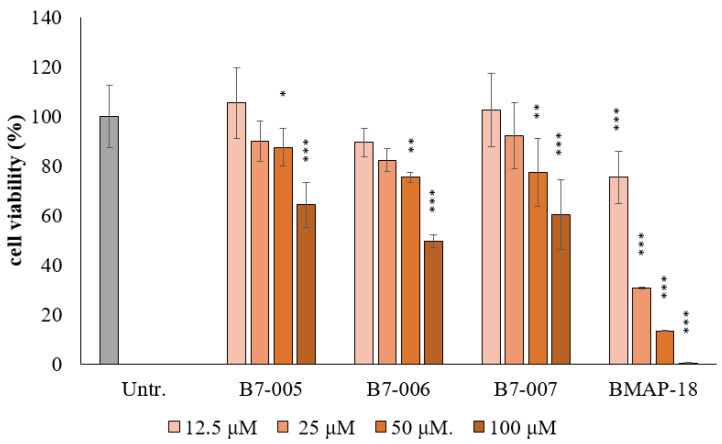
Effects of Peptides on BEAS-2B cell line’s viability. Cell viability was measured using an MTT assay on BEAS-2B cells (4 × 10^5^ cells/mL) after 24 h of exposure to the peptides. Assays were conducted in complete medium (RPMI + 10% FBS, Na-Pyruvate, Pen/Strep, L-Glutamine). Results are presented as percentages of viable cells relative to untreated control (Untr.) cells represented in grey colour (set to 100% viability). Data represent the average ± SD of three independent biological replicates, each performed in triplicate (n ≥ 9). * *p* < 0.05, ** *p* < 0.01, *** *p* < 0.001 compared with the untreated sample (one-way ANOVA test followed by Tukey’s multiple comparisons test).

An MTT assay was used to evaluate cell viability 24 h after exposure to the peptide. Neither B7-005, B7-006, nor B7-007 affected cell viability at antibacterial concentrations (Figure 3), becoming toxic only around 100 µM (B7-006 IC_50_ = 99 µM, B7-007 IC_50_ > 100 µM). In contrast, BMAP-18 was cytotoxic even at low concentrations, with IC_50_ = 17 µM. All B7-peptides exhibited enhanced selectivity and improved safety profiles compared to BMAP-18, as indicated by higher IC_50_ values (>100 µM). The combination of low cytotoxicity and retained antimicrobial activity against *S. aureus* and *P. aeruginosa* resulted in favourable selectivity indices, supporting their ability to effectively target bacteria whilst sparing host cells (Supplementary Table S4).

### 2.4. Peptides Biostability

The biostability of B7-005 and its variants was initially studied in the presence of human elastase and of human sputum naturally containing a mixture of human proteases. For the proteolytic assay, B7-005 and B7-007 were selected. The B7-006 variant was excluded from this analysis as it differs from the parental B7-005 only by the absence of a single Arginine at position 6 (Table 1), a modification that does not introduce any new potential proteolytic cleavage sites. Additionally, the human cathelicidin LL-37 was employed as a reference, being the major endogenous AMP expressed in the human respiratory tract with a key role in the innate defence against pulmonary infections [29,30]. Peptides were incubated with human elastase at a ratio of 1 unit of enzyme per 120 nmol of peptide for 24 h. Aliquots were collected at defined time intervals and analysed by HPLC to monitor peptide degradation (indicated by % of detectable peptide).

**Figure 4:**
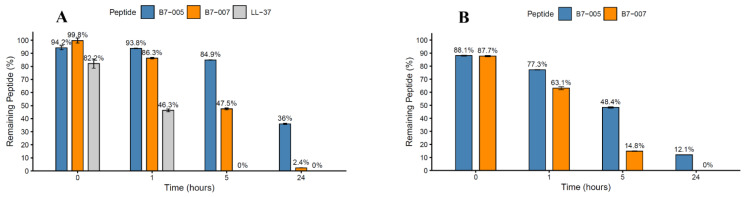
Peptide stability in the presence of human elastase (**A**) or human sputum (**B**). The bars represent the mean area under the curve (%*Area_i_*) from chromatograms of three independent experiments at 0, 1, 5, and 24 h of incubation. Human elastase or human sputum was added to the peptide solutions, and samples for analysis at time = 0 were taken immediately after addition. Error bars indicate the standard deviation (SD).

In the presence of human elastase, both B7-005 and B7-007 remained largely intact after 1 h of incubation, with 94% and 86% of the initial amount still detectable, respectively. However, the percentage of intact peptides declined progressively, with 85% remaining B7-005 after 5 h and 36% after 24 h. Interestingly, B7-007 was degraded a bit faster than its longer variant, with 50% remaining peptide after 5 h and only 2.4% detectable after 24 h (Figure 4A). 

Despite these differences, B7-005 and B7-007 exhibited enhanced resistance to degradation compared to LL-37, which was halved in 1 h and completely disappeared within 5 h. Mass spectrometry of the resulting fragments confirmed the presence of degradation products and allowed some fragments to be identified (Supplementary Figures S2 and S3).

Subsequently, the B7-005 and B7-007 variants were tested in diluted commercial human sputum, where they showed a similar trend. Indeed, both peptides were still largely present after one hour (63–77%), before gradually degrading to become undetectable after 24 h (Figure 4B). Due to the diverse range of proteases in the sputum sample, the resulting degradation profile was more complex (Supplementary Figures S4 and S5).

In both treatments, the most affected site in B7-variants was the Trp-Arg bond, as the corresponding fragments were detected in all samples after one hour of incubation. This susceptibility is likely related to the terminal localization of this bond. Cleavage at the Ile-Arg and Arg-Leu bonds was also observed in all samples incubated for one hour, with the exception of B7-005 incubated with human elastase. This enzyme specificity is not canonical since human neutrophil elastase preferentially cleaves after aliphatic residues such as Val, Ala, and Ile. However, human neutrophil elastase is recognized to exhibit relatively broad substrate specificity [31,32], as evidenced by the identification of fragments generated from cleavage at multiple sites.

Overall, these results suggest that sputum exerts a stronger proteolytic effect, plausibly due to the presence of multiple proteases acting concurrently. In contrast, the Pro-Arg bond appeared resistant to proteolytic cleavage, as did the Arg-Arg bond. Notably, peptide B7-005 contains two additional bonds of this type, and its stability may therefore be associated with the higher abundance of these protease-resistant motifs within its sequence (see Supplementary Figures S2–S5).

## 3. Discussion

The high global mortality associated with multidrug-resistant “priority pathogens” like *P. aeruginosa* and *S. aureus* highlights the need for novel antimicrobial strategies to address respiratory infections.

In this study, we evaluated B7-005 and its shorter variants, B7-006 and B7-007, as candidate AMPs with features potentially suitable for combating pulmonary pathogens.

A key finding of this work is that the antimicrobial potency of B7-006 and B7-007 remains unchanged despite a reduction in size, net positive charge and, in the case of B7-007, a substantial decrease in proline content. The removal of Arg6 in B7-006 and Pro13, and Arg14 in B7-007, did not result in a significant loss of activity against the selected pathogens. This suggests that the “active core” required for function may be smaller than 16 amino acids.

Specifically, B7-007 is only 13 amino acids long and contains only two proline residues, thus it no longer fulfils the formal definition of a PrAMP, which typically requires a proline content of at least 25% [12]. This observation suggests that the minimal pharmacophore required for activity may be more flexible than previously assumed and supports the idea that structural determinants beyond simple proline abundance, such as the specific spatial arrangement of cationic and hydrophobic residues, contribute to biological function.

We previously demonstrated that B7-005, an optimized artificial peptide, derived from the natural PrAMPs Bac7(1–16), is active against all ESKAPEE pathogens [27]. It has also been shown that PrAMPs can exhibit different mechanisms depending on the target bacteria’s ability to internalize the peptide, shifting toward membrane destabilization when protein synthesis inhibition is hindered [27,33].

Here, we observed that further reduction in the size and proline content maintained their antimicrobial activity without macroscopically altering the mode of action compared to the parental B7-005. In fact, all three peptides induced measurable membrane permeabilization in *P. aeruginosa*. Although this level of permeabilization was lower than that observed for the highly lytic peptide BMAP-18 [28], it remained detectable and biologically relevant. However, while membrane perturbation was directly assessed, the intracellular component of the mechanism for the truncated variants may be currently inferred by analogy with the parental B7-005. Given the preservation of the N-terminal ‘active core’ and the high potency observed despite only moderate membrane effects, it is plausible that B7-006 and B7-007 retain the capacity to inhibit protein synthesis.

These results suggest that in *P. aeruginosa*, and potentially in *S. aureus* [27], these variants exert a mechanism of action in which membrane perturbation contributes to the overall effect.

The variants of B7-005 share some structural features with indolicidin, a cathelicidin-derived peptide that, counting only 13-amino-acids, is one of the shortest known natural AMP [34]. Indolicidin is characterized by a high tryptophan content and a net positive charge of +4 (ILPWKWPWWPWRR-NH_2_); it exhibits a broad spectrum of antimicrobial activity through a dual mechanism involving both membrane permeabilization and interaction with intracellular targets [35]. Our variants, B7-006 and B7-007, possess a higher net charge (+7 and +6, respectively) and appear significantly more active than indolicidin against *P. aeruginosa* and *S. aureus* [36]. This aligns with previous studies on indolicidin analogues, where increasing the cationic charge or substituting specific proline residues enhanced antimicrobial potency [36].

Importantly, both variants of B7-005 maintained high activity (12.5–25 µM) across panels of clinical isolates. The narrow distributions of MIC values among the panel and consistent MIC_90_ results suggest limited strain-dependent variability. Furthermore, activity was preserved against MDR phenotypes. Regardless of the specific MDR mechanisms involved, such as target modification, enzymatic inactivation, or efflux systems [37,38], B7-derived peptides remained largely unaffected. This resilience may be attributed to molecular targets that are distinct from those of traditional antibiotics, involving a hybrid mode of action. Although a two-fold increase in MIC was observed against MDR *S. aureus* strains, no such shift was recorded for MDR *P. aeruginosa* strains. Notably, a two-fold variation falls within the expected biological variability of MIC assays and typically does not indicate a meaningful loss of efficacy. In comparison, BMAP-18 showed reduced efficacy against *S. aureus*, highlighting the more balanced spectrum of the B7-derived peptides, a feature that may be advantageous in treating complicated polymicrobial infections.

Regarding safety, B7-005 and its variants showed no significant cytotoxicity toward BEAS-2B bronchial epithelial cells, maintaining a favourable selectivity profile. These peptides exhibited cytotoxic effects only at 50 µM, whereas BMAP-18 demonstrated toxicity at much lower concentration of 12.5 µM. This is particularly relevant as increased hydrophobicity often correlates with higher toxicity to eukaryotic cells [39]. The maintenance of low cytotoxicity suggests that the truncations in B7-006 and B7-007 did not shift the mechanism towards a non-specific lytic action.

Proteolytic stability assays revealed a more nuanced profile. Although B7-007 remained partially intact during the initial incubation with human elastase and sputum, it underwent progressive degradation and was more susceptible to proteolysis than B7-005. This finding highlights a potential trade-off between molecular miniaturisation and protease resistance. The loss of residues that potentially contribute to structural rigidity or protease shielding may render the shortened variants more susceptible to cleavage. This phenomenon has already been observed [40] and it is in line with previous data on fragments of native mammalian PrAMPs, indicating that more extended fragments lasted longer than shorter ones in the aggression of a protease [41]. However, the implications for in vivo efficacy must be contextualized by the kinetics of bacterial killing. While stability is often measured over 24 h, this may not reflect the timeframe required for antimicrobial action; previous studies indicate that B7-005 can exert bactericidal effects within 1–2 h [27]. The presence of intact peptide during these critical early hours is highly relevant; notably, 36% of B7-005 and 2.4% of B7-007 remained detectable after 24 h. Compared to traditional AMPs like LL-37, which may degrade within minutes, the B7 variants demonstrate resilience that suggests they could maintain sufficient integrity to achieve a therapeutic effect in the protease-rich environment of airways.

In summary, this study demonstrates that the artificial B7 scaffold tolerates significant sequence truncation while preserving its primary antimicrobial functions. Our results identify a minimal active core of approximately 13 amino acids, one of the shortest proline- and arginine-containing sequences reported to date, that maintains high potency against both *P. aeruginosa* and *S. aureus*.

The therapeutic interest in these truncated variants is supported by several key factors. First, the B7 scaffold is derived from a class of PrAMPs known to remain active at physiological salt concentrations and within complex biological matrices such as blood and plasma [25]. Second, the favourable safety profile and a satisfactory selectivity index observed here in bronchial epithelial cells are consistent with previous toxicity screenings where the parental B7-005 was found to be non-toxic across four different eukaryotic cell lines [26]. Finally, the maintenance of a hybrid mode of action, combining moderate membrane perturbation with inferred intracellular targeting, allows these variants to bypass common MDR mechanisms in clinical isolates.

While the reduction in size slightly decreases proteolytic stability, the peptides maintain sufficient integrity to exert bactericidal effects within the initial, critical hours of exposure. These findings suggest that B7-006 and B7-007 represent promising lead candidates for further optimization. Future research should focus on defining the structural basis for their ribosomal interaction and evaluating them in vivo pharmacokinetics to fully determine their potential for treating severe respiratory infections.

## 4. Materials and Methods

### 4.1. Peptides

B7-005, B7-006, and B7-007, and BMAP-18 (see Table 1) were produced by NovoPro Bioscience (Shanghai, China) via solid-phase synthesis with F-moc chemistry. Following synthesis, the peptides were purified using RP-HPLC to a purity of >98%. Their molecular identity was subsequently verified by mass spectrometry through determination of their molecular weights. Upon receipt, samples underwent three rounds of lyophilization in 10 mM HCl in order to exchange trifluoroacetate (TFA) with chloride. The peptides were then dissolved in sterile Milli-Q water and quantified using a spectrophotometer (Ultraspec 2100 pro, Amersham Bioscience, Cambridge, UK). Peptide concentrations were determined according to literature methods [42] by calculating molar extinction coefficients with an in-house software tool and measuring absorbance at 214 nm and 280 nm, in accordance with Lambert-Beer’s law. Finally, all peptide solutions were stored at −20 °C.

LL-37 was synthesized by the University of Siena on the automated multiple synthesizer Syro I (MultiSynTech GmbH, Witten, Germany) via solid-phase peptide synthesis using Fmoc chemistry. The protected L-amino acids were provided by Iris Biotech GmbH. The resin was the TentaGel SRAM obtained from Rapp Polymer GmbH (Tübingen, Germany). The crude peptide was purified by HPLC using a reversed-phase C18 column (XBridge peptide BEH C18 Waters, 300 Å, 10 μm, 19 × 250 mm) with 0.1% TFA/water as eluent A and acetonitrile as eluent B. The identity and purity of the peptide were confirmed by reversed-phase chromatography on a Phenomenex Jupiter C18 analytical column (300 Å, 5 μm, 4.6 × 250 mm) and by MALDI-TOF mass spectrometry (ultrafleXtreme, Bruker Daltonics GmbH, Bremen, Germany). Mass spectrometer characterization was done in positive-voltage linear mode. The peptide was stored at −20 °C in lyophilized form.

### 4.2. Bacterial Strains

The reference bacterial strains *Escherichia coli* ATCC 25922, *P. aeruginosa* ATCC 27853, and *P. aeruginosa* ATCC 15692 (PAO1) were originally purchased from American Type Culture Collection (ATCC) (Manassas, VA, USA). Clinical *S. aureus* and *P. aeruginosa* were from the strain collection of Policlinico Umberto I Hospital (Sapienza University of Rome). Antimicrobial susceptibility profiles (Supplementary Tables S2 and S3) were tested using the broth microdilution method by MicroScan WalkAway system (Beckman Coulter, Brea, CA, USA) and interpreted following EUCAST criteria (version 15.0, 2025).

Bacterial strains were stored at −80 °C in glycerol stocks. For all experiments, cultures were propagated in sterile Müller–Hinton broth (MHB) or on Müller–Hinton agar (MHA) plates (Difco Inc., Difco, Becton, Dickinson and Company, Franklin Lakes, NJ, USA). For each assay, bacteria were initially grown overnight (o/n) in 3 mL of MHB at 37 °C under agitation (140 rpm). The following day, these cultures were subcultured by diluting them 1:30 into 10 mL of fresh MHB and incubated at 37 °C with shaking (140 rpm) for approximately 1.5–2.5 h until mid-logarithmic phase was reached (optical density OD_600_ ≈ 0.3). Subsequently, bacterial suspensions were further diluted in fresh MHB to obtain the appropriate cell density required for downstream applications.

### 4.3. Minimum Inhibitory Concentration (MIC)

Minimum inhibitory concentration (MIC) assays were carried out according to Clinical and Laboratory Standards Institute guidelines. Briefly, peptides were initially prepared in MHB at a concentration of 128 µM in a final volume of 100 µL and added to the first wells of a round-bottom 96-well microtiter plate (Sarstedt, Milan, Italy). Two-fold serial dilutions were then performed in MHB by transferring 50 µL of peptide solution into 50 µL of fresh medium. Exponentially growing bacterial cultures (mid-log phase) were diluted in MHB to a final concentration of 1 × 10^6^ CFU/mL. Subsequently, 50 μL of this suspension was dispensed into each well, except for sterility control wells containing only MHB, resulting in a two-fold dilution of both peptide and bacterial concentrations. Plates were sealed with parafilm to limit evaporation and incubated at 37 °C for approximately 18 h. MIC values were defined as the lowest peptide concentration at which no visible bacterial growth was observed. Results are reported as the mode of at least three independent experiments. MIC_90_, defined as the Minimum Inhibitory Concentration required to inhibit the growth of 90% of the specific bacterial isolates tested, was also calculated and reported in the text.

### 4.4. Bacterial Membrane Integrity

Bacterial membrane integrity was evaluated by flow cytometry through the measurement of propidium iodide (PI) uptake, quantified as the mean fluorescence intensity (MFI). Briefly, cultures of *E. coli* ATCC 25922 and *P. aeruginosa* ATCC 27853 were adjusted to 2.5 × 10^5^ CFU/mL in 0.2 μm-filtered MHB and exposed to peptides at their respective MIC values for 1 h at 37 °C. Following treatment, *P. aeruginosa* ATCC 27853 cells were pelleted by centrifugation (16,000× *g*, 5 min) and resuspended in 0.2 μm-filtered PBS. PI was then added to all samples at a final concentration of 10 μg/mL shortly before analysis (5 min). Flow cytometric measurements were performed using an Attune NxT^®^ (ThermoFisher, Carlsbad, CA, USA) equipped with a blue laser (488 nm). Fluorescence signals were collected using BL1 (525/50) and BL3 (695/40) photomultiplier tubes. Data acquisition was carried out on a logarithmic scale, recording signals as area (A). Instrument settings included 310 mV for forward scatter (FSC) and 450 mV for side scatter (SSC), with thresholds set at 5.0 and 2.0, respectively. Hierarchical gating strategies and a flow rate of 200 μL/min were applied to reduce coincident events. Results are reported as mean ± standard deviation (SD) from at least three independent experiments (n = 3).

### 4.5. Cytotoxicity Assay

BEAS-2B cells were obtained from the American Type Culture Collection (ATCC^®^ CRL-3588™, Manassas, VA, USA). Cells were maintained as adherent cultures in complete RPMI medium (EuroClone, Milan, Italy) supplemented with 100 U/mL penicillin, 100 μg/mL streptomycin (Sigma, St. Louis, MO, USA), 2 mM L-glutamine, 1 mM sodium pyruvate (Sigma), and 10% (*v*/*v*, volume/volume) fetal bovine serum (FBS, EuroClone).

Cell viability was assessed using the MTT assay. Briefly, 100 μL of BEAS-2B cell suspension (4 × 10^5^ cells/mL) in complete RPMI were seeded into 96-well flat-bottom plates (EuroClone, Milan, Italy) and incubated overnight at 37 °C in a humidified atmosphere containing 5% CO_2_. The following day, the culture medium was removed and replaced with 100 μL of peptide solutions prepared at the desired concentrations in RPMI.

After 20 h of incubation under the same conditions, 25 μL of MTT solution (5 mg/mL in PBS; Merck Life Science S.r.l., Milan, Italy) was added to each well, followed by a 4-h incubation at 37 °C in the dark. The supernatant was then carefully discarded to avoid disturbing the formazan crystals, and wells were gently washed with 100 μL of sterile PBS. Subsequently, 100 μL of IGEPAL solution (10% *w*/*v* in 10 mM HCl; Merck Life Science S.r.l.) was added to solubilize the crystals, and plates were incubated overnight at 37 °C and 5% CO_2_.

Absorbance was measured at 570 nm using a Nanoquant Infinite M200 Pro plate reader (Tecan, Männedorf, Switzerland). The half-maximal inhibitory concentration (IC_50_) values were calculated by linear interpolation of the dose–response curves. The selectivity index was measured according to Bagla et al. [43].

### 4.6. Peptide Stability

Human Neutrophil Elastase (ELANE) was sourced from Sigma-Aldrich, featuring a molecular weight of 29.5 kDa and an activity of 20 units per milligram. LL-37, B7-005, and B7-007 were solubilised at a final concentration of 1 mg/mL in a 10 mM Tris-HCl buffer, adjusted to pH 7.5. The peptide solutions were incubated with human elastase, using 1 unit of enzyme per 120 nmol of peptide, corresponding to an average peptide-to-enzyme molar ratio of 77:1. The reaction mixture, with a total volume of 130 μL, was then divided into four aliquots, 30 μL each. Human elastase was added to the peptide solutions, and the volumes for analysis at T0 were taken immediately after addition. Human sputum from a normal female individual was purchased from Neo Biotech. B7-005 and B7-007 solutions were diluted in 10 mM Tris-HCl buffer (pH 7.5) at a final concentration of 2 mg/mL. For the reaction setup, a total reaction volume of 130 μL was prepared by mixing equal volumes of the peptide solution and human sputum (50% *v*/*v* final concentration). The peptide-enzyme mixtures were incubated at 37 °C at different times: after 1 h (T1), after 5 h (T5), and after 24 h (T24). At the indicated time intervals, aliquots were withdrawn, diluted with 770 μL of eluent A/B (90% Eluent A: 0.1% trifluoroacetic acid (TFA)–water; 10% Eluent B: Acetonitrile), and analyzed by RP-HPLC (series 200CR3A, PerkinElmer, Waltham, MA, USA). Chromatographic data were processed using TotalChrom Workstation v6.2 (PerkinElmer). The relative abundance of the intact peptides was quantified by area normalization according to the following equation: %*Area_i_* = (*A_i_*/∑*A_total_*) × 100, where “*A_i_*” represents the peak area of the parent peptide and “∑*A_total_*” represents the cumulative area of the detected peaks, assuming unitary response factors at the monitored wavelength (λ = 220 nm). Peak areas were integrated, excluding the injection front. The degradation profile was determined by comparing the relative area at each time point to the initial relative area at T_0_. Molecular weight determination of the proteolytic fragments was performed using a MALDI-TOF mass spectrometer (ultrafleXtreme, Bruker Daltonics GmbH). Analysis was conducted on both the crude reaction mixtures and the predominant peaks collected during RP-HPLC elution. Samples were prepared by mixing 1.5 μL of the sample with 1.5 μL of the HCCA matrix solution. 1.0 μL of the resulting mixture was spotted in triplicate onto a steel MALDI target plate and air-dried at room temperature. The instrument was operated in positive reflectron mode and calibrated using Peptide Calibration Standard (Bruker Daltonics GmbH, Bremen, Germany), covering the mass range 1000–3500 Da. Data processing was managed via FlexAnalysis software v3.4 (Bruker Daltonics GmbH, Bremen, Germany).

### 4.7. Statistical Analysis

The histograms in this work show results as a percentage compared with untreated controls. Data are the mean ± SD of at least three experiments (n ≥ 3) performed in triplicate (n ≥ 9), as indicated in each figure. * *p* < 0.05, ** *p* < 0.01, *** *p* < 0.001 compared with the untreated sample (one-way ANOVA test, GraphPad Prism 10.4.1).

Stability histograms represent the percentage of chromatographic area under the curve, expressed as mean ± SD of three experiments. Statistical analyses and graphical representations were performed using R (version 4.5.2).

## 5. Conclusions

This study identifies a 13-amino-acid minimal antimicrobial motif, among the shortest proline- and arginine-rich sequences reported, that retains potent activity against *P. aeruginosa* and *S. aureus* clinical isolates. Our findings demonstrate that the B7 scaffold is exceptionally resilient to truncation, maintaining a hybrid mechanism of action that bypasses common multidrug resistance (MDR) pathways. While molecular miniaturization leads to a trade-off in proteolytic stability, these variants remain sufficiently intact to exert rapid bactericidal effects. Supported by a favorable safety profile, B7-006 and B7-007 represent promising lead candidates for the future development of compact, mechanistically versatile therapeutics for severe respiratory infections.

## Figures and Tables

**Table 1:** Sequence and features of B7-005 variants and of the alpha-helical peptide BMAP-18.

Peptide	Sequence *	MW	n. Residues	Charge
B7-005	WRIRRRWPRLPRPRWR	2343.8	16	+8
B7-006	WRIRR-WPRLPRPRWR	2186.7	15	+7
B7-007	WRIRR-WPRLPR--WR	1934.3	13	+6
BMAP-18	Ac-GRFKRFRKKFKKLFKKLS-NH_2_ **	2384.0	18	+10

* Amino acids absent in B7-006 or B7-007 are in red color; ** Ac: acetylated; NH_2_: amidated.

**Table 2:** MIC of B7-005 variants on *E. coli* and on 2 different strains of *P. aeruginosa*.

	MIC (µM)		
Bacterial Strain	B7-005	B7-006	B7-007
*E. coli* ATCC 25922	1.56	1.56	3.12
*P. aeruginosa* PAO1 (ATCC 15692)	12.5	12.5	12.5
*P. aeruginosa* ATCC 27823	12.5	12.5	12.5

Results of MIC are the mode of at least three independent experiments (n ≥ 3). MIC was recorded after 18 h of incubation at 37 °C.

## Data Availability

The original contributions presented in this study are included in the article/Supplementary Materials. Further inquiries can be directed to the corresponding authors.

## Supplementary Materials

The following supporting information can be downloaded at: https://www.mdpi.com/article/10.3390/antibiotics15040412/s1, Table S1. MIC of B7-005 variants and BMAP-18 on clinical isolates of *S. aureus* and *P. aeruginosa*; Table S2. Antimicrobial susceptibility profiles of *Staphylococcus aureus* isolates; Table S3. Antimicrobial susceptibility profiles of *Pseudomonas aeruginosa* isolates; Table S4. Selectivity index of peptides B7-005, B7-006, B7-007 and BMAP-18 against clinical isolates of *S. aureus* and *P. aeruginosa* and the reference strains *E. coli* ATCC 25922, *P. aeruginosa* ATCC 27853 and ATCC 15692; Figure S1. Flow cytometric analysis of membrane permeabilization in *P. aeruginosa* ATCC 27853 upon B7-005 (A), B7-006 (B), B7-007 (C) and BMAP-18 (D) treatment; Figure S2. (A) Chromatographic profiles of B7-007 incubated with human elastase incubated for 0 (black), 1 (blue), 5 (green), and 24 (red) hours; Figure S3. (A) Chromatographic profiles of B7-005 incubated with human elastase incubated for 0 (black), 1 (blue), 5 (green), and 24 (red) hours; Figure S4. (A) Chromatographic profiles of B7-007 incubated with human sputum incubated for 0 (black), 1 (blue), 5 (green), and 24 (red) hours; Figure S5. (A) Chromatographic profiles of B7-005 incubated with human sputum incubated for 0 (black), 1 (blue), 5 (green), and 24 (red) hours.

## Abbreviations

AMP	Antimicrobial Peptide
ATCC	American Type Culture Collection
CFU	Colony-Forming Unit
ESKAPE	*Enterococcus faecium*, *Staphylococcus aureus*, *Klebsiella pneumoniae*, *Acinetobacter baumannii*, *Pseudomonas aeruginosa*, *Enterobacter* spp.
ESKAPEE	*Enterococcus faecium*, *Staphylococcus aureus*, *Klebsiella pneumoniae*, *Acinetobacter baumannii*, *Pseudomonas aeruginosa*, *Enterobacter* spp., and *Escherichia* spp.
FBS	Fetal Bovine Serum
FSC	Forward Scatter
HPLC	High-Performance Liquid Chromatography
IC	Inhibitory Concentration
MDR	Multi-Drug Resistant
MFI	Mean Fluorescence Intensity
MHA	Müller–Hinton Agar
MHB	Müller–Hinton Broth
MIC	Minimum Inhibitory Concentration
MTT	Tetrazolium Salt
O/N	Overnight
PI	Propidium Iodide
PMT	Photomultiplier Tubes
PrAMP	Proline-Rich Antimicrobial Peptide
RPM	Revolutions per Minute
RP-HPLC	Reversed-Phase High-Performance Liquid Chromatography
SD	Standard Deviation
SSC	Side Scatter
TFA	Trifluoroacetic Acid

## References

[1] Kumar P., Kizhakkedathu J.N., Straus S.K. (2018). Antimicrobial Peptides: Diversity, Mechanism of Action and Strategies to Improve the Activity and Biocompatibility In Vivo. Biomolecules.

[2] Gera S., Kankuri E., Kogermann K. (2022). Antimicrobial Peptides—Unleashing Their Therapeutic Potential Using Nanotechnology. Pharmacol. Ther..

[3] Oliveira Júnior N.G., Souza C.M., Buccini D.F., Cardoso M.H., Franco O.L. (2025). Antimicrobial Peptides: Structure, Functions and Translational Applications. Nat. Rev. Microbiol..

[4] Rodrigo-Troyano A., Sibila O. (2017). The Respiratory Threat Posed by Multidrug Resistant Gram-Negative Bacteria. Respirology.

[5] Bollar G.E., Keith J.D., Stanford D.D., Oden A.M., Raju S.V., Poore T.S., Birket S.E. (2025). Chronic Coinfection with *Pseudomonas aeruginosa* and Normal Colony *Staphylococcus aureus* Causes Lung Structural Damage in the Cystic Fibrosis Rat. Am. J. Pathol..

[6] Cigana C., Bianconi I., Baldan R., De Simone M., Riva C., Sipione B., Rossi G., Cirillo D.M., Bragonzi A. (2018). Staphylococcus Aureus Impacts Pseudomonas Aeruginosa Chronic Respiratory Disease in Murine Models. J. Infect. Dis..

[7] Sun S. (2025). Emerging Antibiotic Resistance by Various Novel Proteins/Enzymes. Eur. J. Clin. Microbiol. Infect. Dis..

[8] Bahar A.A., Ren D. (2013). Antimicrobial Peptides. Pharmaceuticals.

[9] Scocchi M., Mardirossian M., Runti G., Benincasa M. (2016). Non-Membrane Permeabilizing Modes of Action of Antimicrobial Peptides on Bacteria. Curr. Top. Med. Chem..

[10] Le C.-F., Fang C.-M., Sekaran S.D. (2017). Intracellular Targeting Mechanisms by Antimicrobial Peptides. Antimicrob. Agents Chemother..

[11] Panteleev P.V., Pichkur E.B., Kruglikov R.N., Paleskava A., Shulenina O.V., Bolosov I.A., Bogdanov I.V., Safronova V.N., Balandin S.V., Marina V.I. (2024). Rumicidins Are a Family of Mammalian Host-Defense Peptides Plugging the 70S Ribosome Exit Tunnel. Nat. Commun..

[12] Welch N.G., Li W., Hossain M.A., Separovic F., O’Brien-Simpson N.M., Wade J.D. (2020). (Re)Defining the Proline-Rich Antimicrobial Peptide Family and the Identification of Putative New Members. Front. Chem..

[13] Stączek S., Kunat-Budzyńska M., Cytryńska M., Zdybicka-Barabas A. (2024). Proline-Rich Antimicrobial Peptides from Invertebrates. Molecules.

[14] Krizsan A., Volke D., Weinert S., Sträter N., Knappe D., Hoffmann R. (2014). Insect-Derived Proline-Rich Antimicrobial Peptides Kill Bacteria by Inhibiting Bacterial Protein Translation at the 70 S Ribosome. Angew. Chem. Int. Ed..

[15] Graf M., Mardirossian M., Nguyen F., Seefeldt A.C., Guichard G., Scocchi M., Innis C.A., Wilson D.N. (2017). Proline-Rich Antimicrobial Peptides Targeting Protein Synthesis. Nat. Prod. Rep..

[16] Armas F., Di Stasi A., Mardirossian M., Romani A.A., Benincasa M., Scocchi M. (2021). Effects of Lipidation on a Proline-Rich Antibacterial Peptide. Int. J. Mol. Sci..

[17] Li W., Separovic F., O’Brien-Simpson N.M., Wade J.D. (2021). Chemically Modified and Conjugated Antimicrobial Peptides Against Superbugs. Chem. Soc. Rev..

[18] Shamova O.V., Orlov D.S., Zharkova M.S., Balandin S.V., Yamschikova E.V., Knappe D., Hoffmann R., Kokryakov V.N., Ovchinnikova T.V. (2016). Minibactenecins ChBac7.Nα and ChBac7. Nβ—Antimicrobial Peptides from Leukocytes of the Goat Capra Hircus. Acta Naturae.

[19] Koch P., Schmitt S., Heynisch A., Gumpinger A., Wüthrich I., Gysin M., Shcherbakov D., Hobbie S.N., Panke S., Held M. (2022). Optimization of the Antimicrobial Peptide Bac7 by Deep Mutational Scanning. BMC Biol..

[20] Collins J., McConnell A., Schmitz Z.D., Hackel B.J. (2024). Sequence-Function Mapping of Proline-Rich Antimicrobial Peptides. bioRxiv.

[21] Handley T.N.G., Brakel A., Maxwell A., Ding J., Hadjigol S., D’Costa K., Chandrashekar C., Alder M., Sani M., Mackay G.A. (2025). Developing a Gram-Negative Selective Peptide–Drug Conjugate. ACS Omega.

[22] Kolano L., Knappe D., Berg A., Berg T., Hoffmann R. (2022). Effect of Amino Acid Substitutions on 70S Ribosomal Binding, Cellular Uptake, and Antimicrobial Activity of Oncocin Onc112. ChemBioChem.

[23] Ludwig T., Krizsan A., Mohammed G.K., Hoffmann R. (2022). Antimicrobial Activity and 70S Ribosome Binding of Apidaecin-Derived Api805 with Increased Bacterial Uptake Rate. Antibiotics.

[24] Skowron K.J., Baliga C., Johnson T., Kremiller K.M., Castroverde A., Dean T.T., Allen A.C., Lopez-Hernandez A.M., Aleksandrova E.V., Klepacki D. (2023). Structure–Activity Relationships of the Antimicrobial Peptide Natural Product Apidaecin. J. Med. Chem..

[25] Mardirossian M., Sola R., Beckert B., Valencic E., Collis D.W.P., Borišek J., Armas F., Di Stasi A., Buchmann J., Syroegin E.A. (2020). Peptide Inhibitors of Bacterial Protein Synthesis with Broad Spectrum and SbmA-Independent Bactericidal Activity Against Clinical Pathogens. J. Med. Chem..

[26] Di Stasi A., Bozzer S., Pacor S., de Pascale L., Morici M., Favero L., Spazzapan M., Pegoraro S., Bulla R., Wilson D.N. (2024). The Proline-Rich Antimicrobial Peptide B7-005: Low Bacterial Resistance, Safe for Human Cells and Effective in Zebrafish Embryo Bacteraemia Model. Open Biol..

[27] Di Stasi A., Capolla S., Morici M., Bozzer S., Berger M., Pacor S., Pham T.D., Spurio R., Fabbretti A., Macor P. (2025). Mechanistic Divergence and Differential Antibacterial Potency of the Proline-Rich Antimicrobial Peptide B7-005 Across ESKAPE + E Pathogens. Probiotics Antimicro. Prot..

[28] Jahan I., Kumar S.D., Shin S.Y., Lee C.W., Shin S.-H., Yang S. (2023). Multifunctional Properties of BMAP-18 and Its Aliphatic Analog against Drug-Resistant Bacteria. Pharmaceuticals.

[29] Dürr U.H.N., Sudheendra U.S., Ramamoorthy A. (2006). LL-37, the Only Human Member of the Cathelicidin Family of Antimicrobial Peptides. Biochim. Biophys. Acta (BBA)—Biomembr..

[30] Fahy R.J., Wewers M.D. (2005). Pulmonary Defense and the Human Cathelicidin hCAP-18/LL-37. Immunol. Res..

[31] Kasperkiewicz P., Poreba M., Snipas S.J., Parker H., Winterbourn C.C., Salvesen G.S., Drag M. (2014). Design of Ultrasensitive Probes for Human Neutrophil Elastase through Hybrid Combinatorial Substrate Library Profiling. Proc. Natl. Acad. Sci. USA.

[32] Fu Z., Thorpe M., Akula S., Chahal G., Hellman L.T. (2018). Extended Cleavage Specificity of Human Neutrophil Elastase, Human Proteinase 3, and Their Distant Ortholog Clawed Frog PR3—Three Elastases with Similar Primary but Different Extended Specificities and Stability. Front. Immunol..

[33] Runti G., Benincasa M., Giuffrida G., Devescovi G., Venturi V., Gennaro R., Scocchi M. (2017). The Mechanism of Killing by the Proline-Rich Peptide Bac7(1-35) Against Clinical Strains of Pseudomonas Aeruginosa Differs from That Against Other Gram-Negative Bacteria. Antimicrob. Agents Chemother..

[34] Ioannou P., Baliou S., Kofteridis D.P. (2023). Antimicrobial Peptides in Infectious Diseases and Beyond—A Narrative Review. Life.

[35] Batista Araujo J., Sastre de Souza G., Lorenzon E.N. (2022). Indolicidin Revisited: Biological Activity, Potential Applications and Perspectives of an Antimicrobial Peptide Not yet Fully Explored. World J. Microbiol. Biotechnol..

[36] Ngo Van H., Luong Xuan H., Le Viet H., Phuong H.B.T., Do Hai Y., Thang N.Q., Truong Thanh T., Yen T.V., Minh T.N., Van L.N. (2025). Indolicidin Derivatives as Potent Dual-Action Antifungal and Antibacterial Agents for the Treatment of Skin Infections: A Comprehensive Study from In Vitro to In Vivo Evaluation. PLoS ONE.

[37] Alharbi M.S., Moursi S.A., Alshammari A., Aboras R., Rakha E., Hossain A., Alshubrumi S., Alnazha K., Khaja A.S.S., Saleem M. (2025). Multidrug-Resistant Pseudomonas Aeruginosa: Pathogenesis, Resistance Mechanisms, and Novel Therapeutic Strategies. Virulence.

[38] Shao K., Yang Y., Gong X., Chen K., Liao Z., Ojha S.C. (2025). Staphylococcal Drug Resistance: Mechanisms, Therapies, and Nanoparticle Interventions. IDR.

[39] Chen Y., Guarnieri M.T., Vasil A.I., Vasil M.L., Mant C.T., Hodges R.S. (2007). Role of Peptide Hydrophobicity in the Mechanism of Action of Alpha-Helical Antimicrobial Peptides. Antimicrob. Agents Chemother..

[40] Checco J.W., Lee E.F., Evangelista M., Sleebs N., Rogers K.L., Pettikiriarachchi A., Kershaw N.J., Eddinger G.A., Belair D.G., Wilson J.L. (2015). α/β-Peptide Foldamers Targeting Intracellular Protein–Protein Interactions with Activity in Living Cells. J. Am. Chem. Soc..

[41] Mattiuzzo M., Gobba C.D., Runti G., Mardirossian M., Bandiera A., Gennaro R., Scocchi M. (2014). Proteolytic Activity of Escherichia Coli Oligopeptidase B Against Proline-Rich Antimicrobial Peptides. J. Microbiol. Biotechnol..

[42] Kuipers B.J.H., Gruppen H. (2007). Prediction of Molar Extinction Coefficients of Proteins and Peptides Using UV Absorption of the Constituent Amino Acids at 214 Nm to Enable Quantitative Reverse Phase High-Performance Liquid Chromatography-Mass Spectrometry Analysis. J. Agric. Food Chem..

[43] Bagla V.P., McGaw L.J., Elgorashi E.E., Eloff J.N. (2014). Antimicrobial Activity, Toxicity and Selectivity Index of Two Biflavonoids and a Flavone Isolated from Podocarpus Henkelii (Podocarpaceae) Leaves. BMC Complement. Altern. Med..

