# Supplementary material for: Two Shorter Variants of the Proline-Rich Antimicrobial Peptide B7-005 Scaffold Active Against Clinical Isolates of Pseudomonas aeruginosa and Staphylococcus aureus
Source: Antibiotics (Basel). 2026 Apr 18;15(4):412. doi: 10.3390/antibiotics15040412 (PMC13114054; doi:10.3390/antibiotics15040412)
Supplement: Supplementary file 1 [file antibiotics-15-00412-s001.zip › antibiotics-4211758-supplementary.pdf]

Supplementary Table S1. **MIC of B7-005 variants and BMAP-18 on clinical isolates of *S. aureus* and *P. aeruginosa.***

		MIC (μM)			
**Bacterial strains**		**B7-005**	**B7-006**	**B7-007**	**BMAP-18**
S. aureus	SA01	6.25	6.25	6.25	6.25
	SA02	6.25	3.12	3.12	>100
	SA03	25	12.5	25	50
	SA04	6.25	3.12	6.25	6.25
	SA05	6.25	6.25	6.25	6.25
	SA06 *	12.5	25	12.5	50
	SA07 *	12.5	12.5	12.5	25
	SA08 *	12.5	12.5	12.5	25
	SA09 *	25	25	12.5	25
	SA10 *	12.5	12.5	12.5	25
P. aeruginosa	PA01	50	12.5	12.5	3.12
	PA02	25	12.5	25	12.5
	PA03	12.5	6.25	12.5	6.25
	PA04	25	12.5	12.5	1.56
	PA05	12.5	12.5	12.5	6.25
	PA06 *	25	25	25	3.12
	PA07 *	12.5	12.5	12.5	0.78
	PA08 *	25	25	12.5	6.25
	PA09 *	12.5	25	25	6.25
	PA10 *	12.5	6.25	12.5	1.56

*Multi-drug resistant (MDR) phenotype : resistant to at least three out of the eleven different antibiotic classes tested
The MIC values, expressed in μM, are the mode of at least three independent experiments (n = 3). MIC was recorded after 18h of incubation at 37°C.

Supplementary Table S2. Antimicrobial susceptibility profiles of *Staphylococcus aureus* isolates

Bacterial strains	OXA	CPT	LVX	CLI	ERY	GEN	RIF	SXT	VAN	LZD	DAP
SA01	S	S	I	S	S	S	S	S	S	S	S
SA02	S	S	I	R	R	S	S	S	S	S	S
SA03	S	S	I	S	S	S	S	S	S	S	S
SA04	S	S	I	R	R	S	S	S	S	S	S
SA05	S	S	I	R	R	S	S	S	S	S	S
SA06*	R	S	R	R	S	S	S	S	S	S	S
SA07*	R	S	R	R	R	S	S	S	S	S	S
SA08*	R	S	I	R	R	S	S	S	S	S	S
SA09*	R	S	R	R	R	S	S	S	S	S	S
SA10*	R	S	R	R	R	S	S	S	S	S	S

Abbreviations: OXA, oxacillin; CPT, ceftaroline; LVX, levofloxacin; CLI, clindamycin; ERY, erythromycin; GEN, gentamicin; RIF, rifampicin; SXT, trimethoprim/sulfamethoxazole; VAN, vancomycin; LZD, linezolid; DAP, daptomycin.
Susceptibility categories: S, susceptible; I, susceptible, increased exposure; R, resistant. Susceptibility was interpreted according to EUCAST clinical breakpoints (version 15.0, 2025).

*Multi-drug resistant (MDR) phenotype: resistant to at least three out of the eleven different antibiotic classes tested

Supplementary Table S3. Antimicrobial susceptibility profiles of *Pseudomonas aeruginosa* isolates

Bacterial strains	MEM	IPM	FEP	CAZ	CZA	C/T	TZP	CIP	AMK	TOB	CST
PA01	S	I	I	I	S	S	I	I	S	S	S
PA02	S	I	I	I	S	S	I	I	S	S	S
PA03	S	I	I	I	S	S	I	I	S	S	S
PA04	S	I	I	I	S	S	I	I	S	S	S
PA05	S	I	I	I	S	S	I	I	S	S	S
PA06*	R	R	R	R	R	R	R	R	R	R	S
PA07*	R	R	R	R	R	R	I	R	R	R	S
PA08*	R	R	R	R	R	R	R	I	S	S	S
PA09*	R	R	R	R	S	S	R	I	S	S	S
PA10*	R	I	I	I	S	S	R	R	S	R	S

Abbreviations: MEM, Meropenem; IPM, Imipenem; FEP, Cefepime; CAZ, Ceftazidime; CZA, Ceftazidime/Avibactam; C/T, Ceftolozane/Tazobactam; TZP, Piperacillin/Tazobactam; CIP, Ciprofloxacin; AMK, Amikacin; TOB, Tobramycin; CST, Colistin.
Susceptibility categories: S, susceptible; I, susceptible, increased exposure; R, resistant. Susceptibility was interpreted according to EUCAST clinical breakpoints (version 15.0, 2025).

*Multi-drug resistant (MDR) phenotype: resistant to at least three out of the eleven different antibiotic classes tested

Supplementary Table S4. Selectivity index of peptides B7-005, B7-006, B7-007 and BMAP-18 against clinical isolates of *S. aureus* and *P. aeruginosa* and the reference strains *E. coli* ATCC 25922, *P. aeruginosa* ATCC 27853 and ATCC 15692.

		selectivity index			
		**B7-005**	**B7-006**	**B7-007**	**BMAP-18**
	IC_{50} (µM)	**>100**	**99.7**	**>100**	**19.6**
**Bacterial strains**					
S. aureus	SA01	32.0	15.9	32.0	3.1
	SA02	32.0	31.9	64.1	
	SA03	8.0	8.0	8.0	0.4
	SA04	32.0	31.9	32.0	3.1
	SA05	32.0	15.9	32.0	3.1
	SA06*	16.0	4.0	16.0	0.4
	SA07*	16.0	8.0	16.0	0.8
	SA08*	16.0	8.0	16.0	0.8
	SA09*	8.0	4.0	16.0	0.8
	SA10*	16.0	8.0	16.0	0.8
P. aeruginosa	PA01	4.0	8.0	16.0	6.3
	PA02	8.0	8.0	8.0	1.6
	PA03	16.0	15.9	16.0	3.1
	PA04	8.0	8.0	16.0	12.6
	PA05	16.0	8.0	16.0	3.1
	PA06*	8.0	4.0	8.0	6.3
	PA07*	16.0	8.0	16.0	25.1
	PA08*	8.0	4.0	16.0	3.1
	PA09*	16.0	4.0	8.0	3.1
	PA10*	16.0	15.9	16.0	12.6
E. coli	ATCC 25922	128.2	63.9	64.1	
P. aeruginosa	ATCC 15692	16	8.0	16	
P. aeruginosa	ATCC 27853	16	8.0	16	

Selectivity index (SI) calculated as the ratio between IC_{50} (µM) and MIC (µM) for each condition [1]. For peptides B7-005 and B7-007, for which the IC_{50} was >100 µM (the highest concentration tested) the SI was calculated assuming an IC_{50} of 200 µM.

1. Bagla, V.P.; McGaw, L.J.; Elgorashi, E.E.; Eloff, J.N. Antimicrobial Activity, Toxicity and Selectivity Index of Two Biflavonoids and a Flavone Isolated from Podocarpus Henkelii (Podocarpaceae) Leaves. *BMC Complement Altern Med* **2014**, *14*, 383, doi:10.1186/1472-6882-14-383.

*Multi-drug resistant (MDR) phenotype: resistant to at least three out of the eleven different antibiotic classes tested

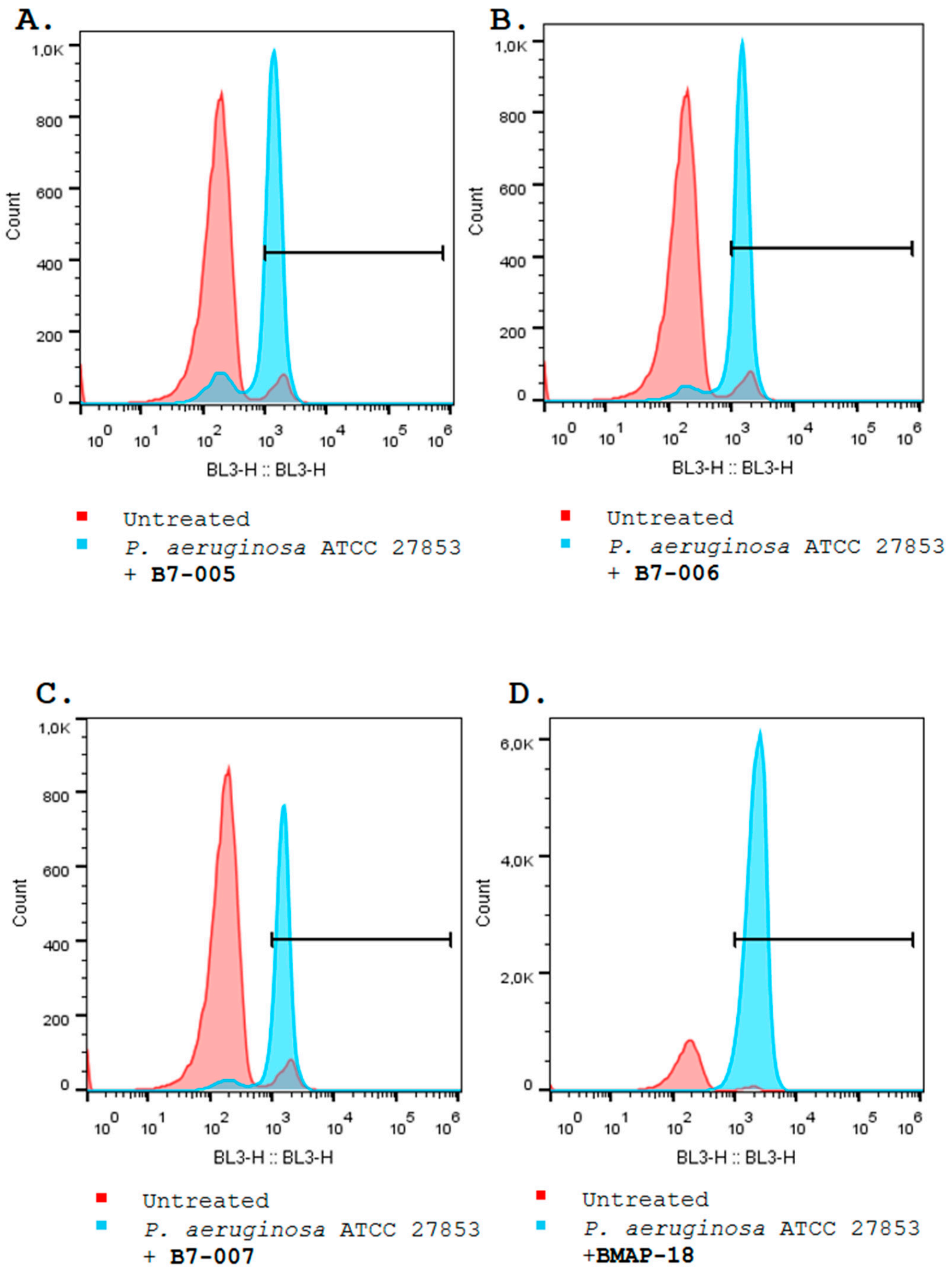

Supplementary Figure S1: Flow cytometric analysis of membrane permeabilization in *P. aeruginosa* ATCC 27853 upon B7-005 (**A**), B7-006 (**B**), B7-007 (**C**) and BMAP-18 (**D**) treatment. Propidium iodide (PI) fluorescence (BL3-H, log scale) is shown as event count. Peptide-treated cells (blue) exhibit a fluorescence shift compared to untreated controls (red). The black bar denotes the PI-positive gate.

A

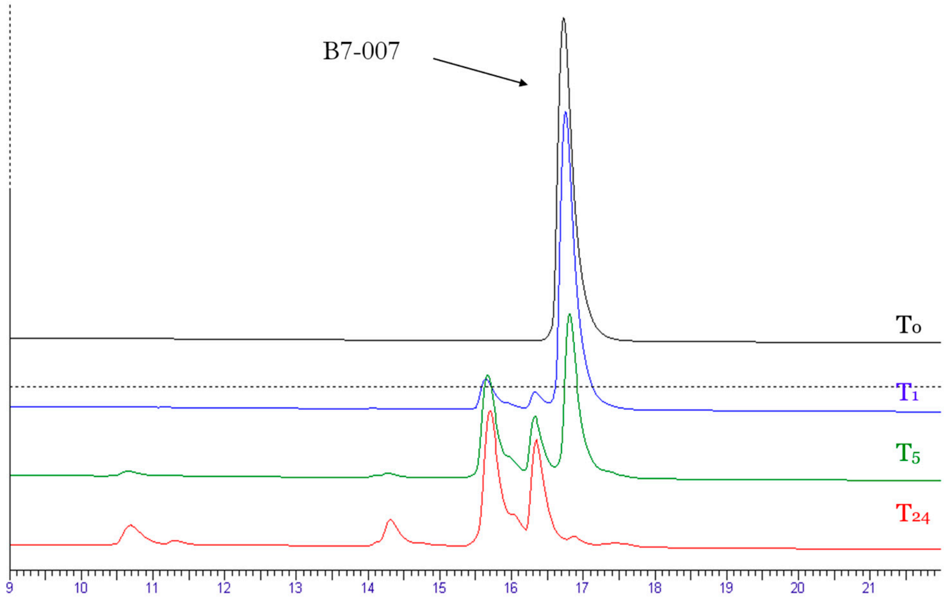

B WRIRRWPRLPRWR

Sequence	MW
WRIRRWPRLPRWR	1934 Da
RRWPRLPRWR	1479 Da
RRWPRLPR	1136 Da
RIRRWPRL	1153 Da
WPRLPRWR	1166 Da
RWPRLPRW	

Supplementary Figure S2. **(A)** Chromatographic profiles of B7-007 incubated with human elastase incubated for 0 (black), 1 (blue), 5 (green), and 24 (red) hours. The degradation profile was determined by comparing the relative area at each time point to the initial relative area at T_0 as described in the material and methods section. For example: the area under the curve of B7-007 at T_{24} is 2.4 % relative to the T_0. The main peaks identified at T_{24} with retention times of 10.6, 14.2, 15.6 and 16.3 min were analysed by MALDI-TOF spectrometry. **(B)** Sequence of B7-007 and list of its degradation fragments identified by MALDI-TOF mass spectrometry. Putative cleavage sites of elastase are indicated by the black arrows. The intact peptide was also detected and confirmed at 1934 Da. The sequences in italics, corresponding to the m/z 1166 peak, can be attributed to two different peptide fragments.

A

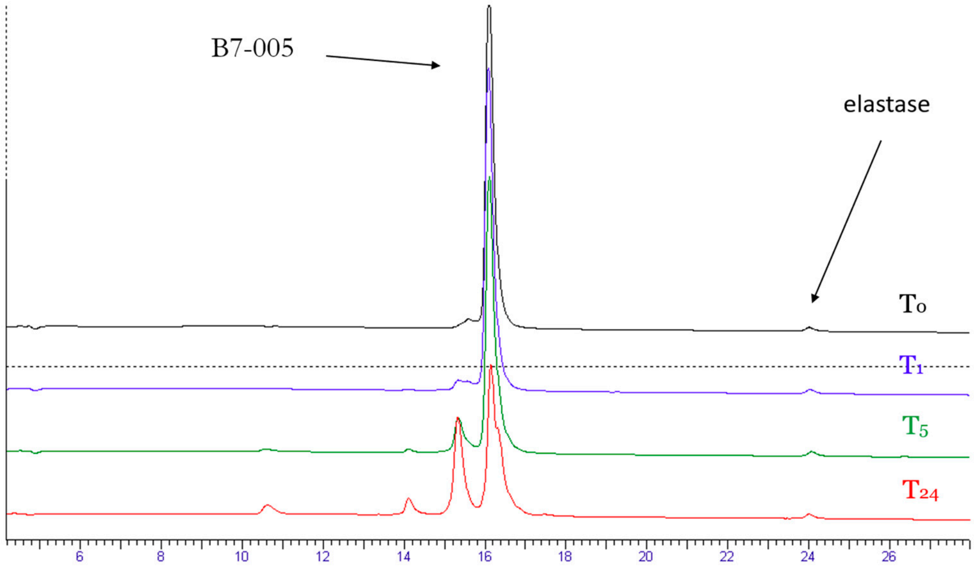

B

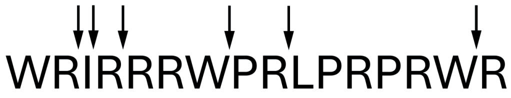

Sequence	MW
WRIRRRWPRLPRPRWR	2344 Da
IRRRWPRLPRPRWR	2002 Da
RRRWPRLPRPRWR	1888 Da
RRWPRLPRPRWR	1732 Da
PRLPRPRWR	1234 Da
LPRPRW	824 Da
IRRRW	786 Da
WRIRRR	*942 Da*
RIRRRW	

Supplementary Figure S3. **(A)** Chromatographic profiles of B7-005 incubated with human elastase incubated for 0 (black), 1 (blue), 5 (green), and 24 (red) hours. The degradation profile was determined by comparing the relative area at each time point to the initial relative area at T_0 as described in the material and methods section. For example: the area under the curve of B7-005 at T_{24} is 36% relative to the T_0. The main peaks identified at T_{24} with retention times of 10.6, 14.1, 15.3 and 16.3 min were analysed by MALDI-TOF spectrometry. **(B)** Sequence of B7-005 and list of its degradation fragments identified by MALDI-TOF mass spectrometry. Putative cleavage sites of elastase are indicated by the black arrows. The sequences in italics, corresponding to the m/z 942 peak, can be attributed to either the 1–6 or the 2–7 peptide fragments.

A

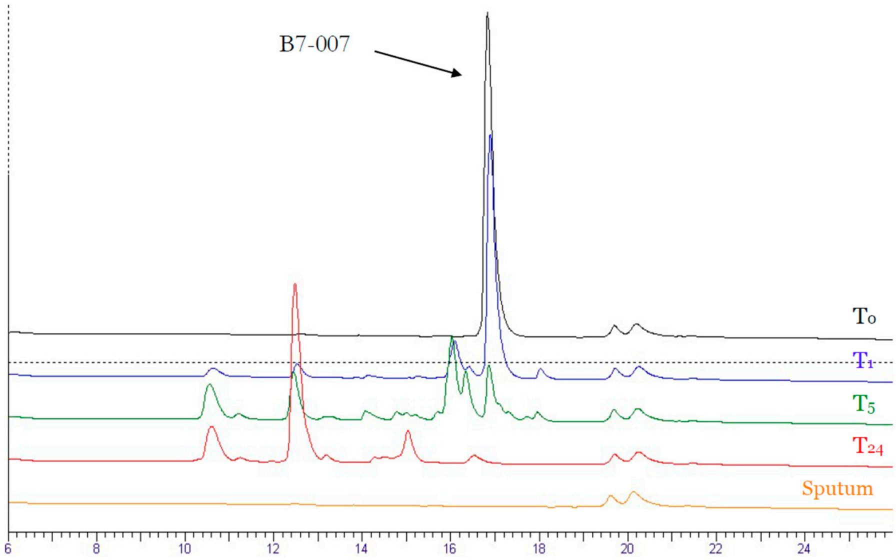

B

WRIRRWPRLPRWR

Sequence	MW
RRWPRLPR	1136 Da
WPRLPRWR	1166 Da
RWPRLPRW	
PRLPRWR	980 Da
RWPRLPR	
RRWPRLP	
RLPRWR	883 Da
RRWPRL	
IRRWPR	
RIRRWP	
PRLPRW	824 Da
WPRLPR	
RWPRLP	

Supplementary Figure S4. (A) Chromatographic profiles of B7-007 incubated with human sputum incubated for 0 (black), 1 (blue), 5 (green), and 24 (red) hours. The degradation profile was determined by comparing the relative area at each time point to the initial relative area at T_0 as described in the material and methods section. The orange line represents the background signal to the sputum alone. The main peaks identified at T_{24} with retention times of 10.6, 12.4, 15.0 and 16.5 min were analysed by MALDI-TOF spectrometry (**B**) Sequence of B7-007 and list of its degradation fragments identified by MALDI-TOF mass spectrometry. Putative cleavage sites of elastase are indicated by the black arrows. Note that several fragments share identical molecular weights; consequently, only the 1136 Da fragment could be confidently assigned to the indicated cleavage sites. The sequences in italics correspond to isobaric fragments that cannot be uniquely assigned to a single cleavage event.

A

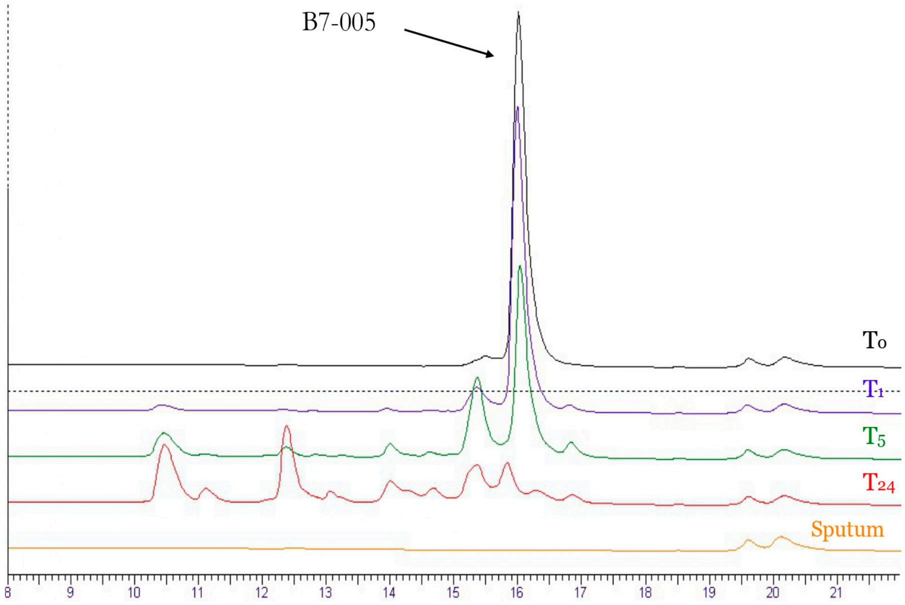

B

WRIRRRWPRLPRPRWR

Sequence	MW
WRIRRRWPRLPRPRWR	2344 Da
WRIRRRWPRLPRPRWR	2188 Da
RRRWPRLPRPRWR	1888 Da
RIRRRWPRLPR	1562 Da
RRRWPRLPRPR	1546 Da
RLPRPR	794 Da
IRRRW	786 Da
RIRRR	756 Da
RIRRRWPRLPRP	1659 Da
IRRRWPRLPRPR	
RRRWPRLP	1136 Da
RRWPRLPR	
WRIRRR	942 Da
RIRRRW	

Supplementary Figure S5. (A) Chromatographic profiles of B7-005 incubated with human sputum incubated for 0 (black), 1 (blue), 5 (green), and 24 (red) hours. The degradation profile was determined by comparing the relative area at each time point to the initial relative area at T_0 as described in the material and methods section. The orange line represents the background signal to the sputum alone. The main peaks identified at T_{24} with retention times of 10.4, 11.1, 12.8, 14.0, 15.3 and 15.8 min were analysed by MALDI-TOF spectrometry **(B)** Sequence of B7-007 and list of its degradation fragments identified by MALDI-TOF mass spectrometry. Putative cleavage sites of elastase are indicated by the black arrows. The sequences in italics correspond to isobaric fragments that cannot be uniquely assigned to a single cleavage event.
